# New Appliances and Things Medical

**Published:** 1899-11-04

**Authors:** 


### NEW APPLIANCES AND THINGS MEDICAL.

### M.D. CARBOLIC SOAP AND THE CHANCELLOR No. 1 SOAP.

(Chancellor, Walker, and Wallis, Limited, London and Hull.)

M.D. carbolic soap appears a well-made and economical soap impregnated with carbolic acid. It should be found valuable in households for maintaining that condition of domestic hygiene which is necessary to health. If all floors, woodwork, clothing, bedding, &c., were washed with this economical soap, the chances of illness through contagious and dirt diseases would be reduced to a minimum. In crowded tenements and cottages of the poorer description its systematic use would save much distress and misery. The Chancellor soap is a capital soap for ordinary purposes. The manufacturers make the special claim for it that it will soften the skin and prevent chapped hands, and for this reason it should be particularly appreciated by the home laundress.

### NUTRIENT POWDER.

(Brand and Co., Limited, 11, Little Stanhope Street, Mayfair, London, W.)

The above nutrient powder (Dence's patent) is prepared from raw meat from which the moisture has been removed at a temperature below that at which the muscle proteids coagulate. It is completely sterilised and tasteless, and contains all the constituents of lean meat in an unaltered condition. It is equivalent to four times its weight of fresh lean meat. As a nutritive agent it is manifestly superior to the usual meat extracts, essences, and the like, and should be specially serviceable in the treatment of those conditions where a proteid diet is enjoined, but where the food must be of a digestible character, concentrated in form, and unlikely to leave any débris in the bowels.

